# Apraxia as a Predictor of Poststroke Recovery: Insights From the Birmingham Cognitive Screening Program

**DOI:** 10.1161/STROKEAHA.125.051414

**Published:** 2025-10-07

**Authors:** Elisabeth Rounis, Siddharth Ramanan, Wai-Ling Bickerton, Nele Demeyere, Matthew A. Lambon Ralph

**Affiliations:** 1 Medical Research Council Cognition and Brain Sciences Unit, University of Cambridge, United Kingdom (E.R., S.R., M.A.L.R.).; 2 Department of Brain Sciences, Faculty of Medicine, Imperial College London, United Kingdom (E.R.).; 3 School of Psychology University of Birmingham, United Kingdom (W.-L.B.).; 4 Nuffield Department of Clinical Neurosciences, University of Oxford, Oxford, United Kingdom (N.D.).

**Keywords:** activities of daily living, apraxias, gesture, morbidity, stroke

## Abstract

**BACKGROUND:**

Limb apraxia is common after stroke and may affect long-term activities of daily living. This study investigates whether early subacute limb praxis scores predict long-term activities of daily living outcomes over and above other cognitive deficits.

**METHODS:**

This longitudinal observational study analyzed data from the BCoS (Birmingham Cognitive Screen) cohort, conducted between 2010 and 2015 across multiple stroke centers in the United Kingdom. Two-hundred-fifty-six first-ever computed tomography confirmed stroke survivors (56.3% men; mean±SD age=68.3±11.4 y) were assessed <1 month poststroke (subacute) and >9 months (chronic). BCoS cohort comprises 34 cognitive tasks and 4 assess limb praxis. Scores were rescaled to 0 to 100. Functional outcome was the 20-point Barthel index (BI). Stepwise multiple linear regression with 4-fold internal cross-validation tested whether subacute cognitive and praxis performance predicted chronic BI, adjusting for baseline BI, and other cognitive domains.

**RESULTS:**

Mean BI improved from 13.3±5.5 to 17.3±3.9. Higher subacute limb praxis scores predicted better chronic activities of daily living: gesture production β=–0.0555 (*P*=0.0008), gesture recognition β=–0.0349 (*P*=0.017), meaningless gesture imitation β=0.0338 (*P*=0.0047). The full model explained 60% of BI variance and outperformed a model without praxis measures (ΔR²=0.04; ANOVA *P*<0.001).

**CONCLUSIONS:**

Detailed early limb praxis testing adds independent prognostic value for long-term activities of daily living and should be incorporated into routine poststroke assessments to target rehabilitation.

Stroke is the global leading cause of morbidity. Despite improvements in mortality rates with advanced acute stroke treatments, such as thrombolysis and thrombectomy, two-thirds of stroke survivors still leave UK hospitals with a disability.^1^ Emerging evidence shows that cognitive deficits can help predict poststroke recovery. Among these deficits, limb apraxia emerges as a consequence of stroke, which is linked to prolonged disability and dependence.^2,3^ This disorder impedes learned or skilled actions in the absence of basic motor execution impairments. It manifests as difficulty with everyday tasks, such as combing hair, dressing, and preparing a cup of coffee. In more severe cases, these challenges may extend to using utensils, such as shaving or cutting with knives, placing individuals at risk if apraxia is overlooked. The intricate demands of neuropsychological testing coupled with the diverse manifestations of apraxia complicate the systematic assessment of patients’ deficits in clinical settings. Consequently, formulating targeted neuro-rehabilitative or compensatory strategies becomes challenging, with patients frequently leaving acute stroke units uninformed about their condition and lacking suitable management strategies.

Few studies encompass a comprehensive array of neuropsychological assessments necessary to identify apraxia and other cognitive deficits poststroke, aligning them with stroke outcome measures. Moreover, there are very few longitudinal studies investigating how complex neuropsychological deficits may recover over time and whether they lead to worse outcomes. Cognitive difficulties following a stroke are typically evaluated through either brief yet somewhat constrained general tests or exhaustive but time-consuming assessments that focus on specific domains, such as language, memory, neglect, and praxis that are difficult to deliver in the acute setting.

Addressing these challenges, we leverage the BCoS study (Birmingham Cognitive Screen) data set, which uniquely comprised both broad and shallow neuropsychological sampling of multiple cognitive abilities, including praxic deficits, in a time-effective manner, designed to be inclusive for patients with aphasia and spatial neglect.^4^

## Methods

### Data Availability Statement

Due to patient privacy restrictions, the original video recordings of praxis assessments cannot be shared publicly. However, anonymized cognitive and functional outcome scores are available from the first author on reasonable request, subject to ethical approvals.

#### Participants and Measures

This study utilized data from participants with stroke enrolled in the BCOS cohort study, conducted between 2010 and 2015 across multiple stroke centers in the United Kingdom.^4^ Eligible participants had a first computed tomography confirmed stroke, were able to provide informed consent and had no prior neurological or psychiatric conditions. Participants were recruited from multiple centers to reduce selection bias. Cognitive assessments were administered by trained raters using standardized protocols. All gave written informed consent under approved Essex ethics procedures (08/H0301/6. 2011/2012).

Of the original cohort of over 424 patients, a subset of 256 patients (56.3% male and 43.7% female; mean age, 68.3; SD, 11.4) who had a first computed tomography confirmed and completed the full battery of BCoS cognitive measures-including all cognitive tasks all apraxia tasks and Barthel index (BI) activities of daily living (ADL) measures at both time points (early subacute^5^: <1 month; chronic: >9 month) were included. Stroke types included ischemic (73%), hemorrhagic (18.8%), and undetermined (8%). Figure S1 illustrates the data analysis flowchart for participant recruitment, follow-up, and exclusions. This was, therefore, a complete case analysis with no missing data across key variables included in the primary analysis, in accordance with Strengthening the Reporting of Observational Studies in Epidemiology recommendations. Participant characteristics for included versus excluded individuals are provided in the Supplemental Material; Tables S1 through S3. Comparison of included and excluded participants demonstrated no significant differences in age, sex, or stroke type (see Table S3).

#### Neuropsychological Assessments

Cognitive performance was assessed using the BCoS, which includes 34 tasks across domains, such as executive function (4 measures), memory (3 measures), language (7 measures), calculation (3 measures), visuo-spatial attention (3 measures), tactile extinction (8 measures), and orientation (2 measures). Among these were 4 praxis tasks, described below,^6^ and 1 constructional apraxia task, reflecting visuospatial and motor integration ability. All tasks were videotaped and scored using published criteria.^6–8^ To enable comparison across measures, scores were standardized to a 0 to 100 scale. Table 1 shows the final patient demographic/stroke characteristics and scores for apraxia tasks.

**Table 1.**
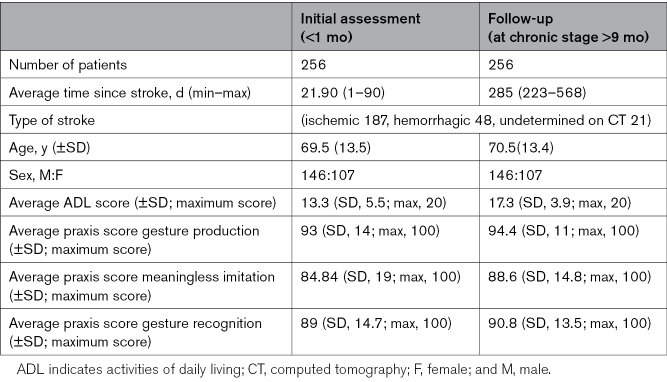
Patient Demographic Details, Stroke Severity Based on Their ADL Score and Praxis Results (SD)

### Limb Praxis Tasks

1. Meaningless gesture imitation involving imitation of meaningless 8 finger and 4 hand gestures adapted from De Renzi et al^7^—the total score for this task was 12.
2. Gesture production: requiring participants to pantomime 3 transitive (object –related) gestures (eg, showing how to use a hammer, glass, and salt cellar) and 3 intransitive gestures (eg, hitch-hiking, military salute, and stopping) to auditory/written words input adapted from the Florida Apraxia Task^8^—the total score for this task was 6.
3. Gesture recognition: a forced choice recognition of pantomimes done by the examiner involving 3 transitive (eg, using a cup, key, or lighter) and 3 intransitive (eg, waving goodbye, good and come over) gestures adapted from Peigneux et al 2000^9^—the total score for this task was 6.
4. Multistep object use task: patients had to put the batteries in a torch in the correct orientation to light it, the total score for this task was 12.

The BI-ADL was used to measure functional outcome at both time points. We used the 20-point version of the BI, which assesses independence in 10 daily activities (eg, mobility, toileting, feeding) with higher scores indicating greater functional independence.^10^

Sample size calculations using the pwrss package in R (repeated ANOVA model, with 2 repeated time measures and correlation between repeated measures=0.5) suggested a minimum N of 33 for a detecting statistically significant effects (*P*<0.05) with medium effect size (eta-square = 0.06) at 80% power (Table 2).

**Table 2.**
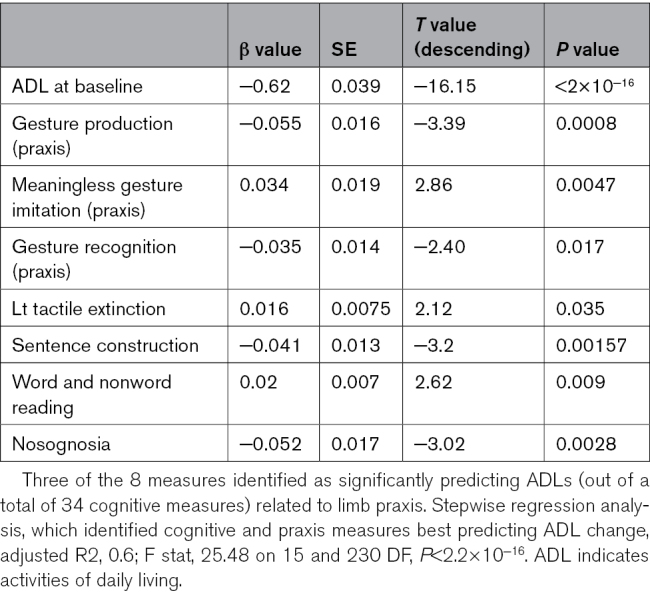
Most Significant Predictors of ADL Change (Stepwise Linear Regression AIC=602.24, Rsquared=0.30 F statistic 8.553 on 14 and 232 DF, *P*=7.54×10^−15^)

#### Statistical Analyses

Statistical analyses were conducted in RStudio v.4.2.0. We used multivariate predictive forward stepwise linear regression modeling to predict the change in ADL score between the chronic (>9 months) and subacute (<1 month) stages using cognitive and limb praxis measured at the subacute stage. All models were internally validated using 4-fold cross-validation per Transparent Reporting of a Multivariable Prediction Model for Individual Prognosis or Diagnosis guidelines.^11^ A sensitivity analysis was conducted to assess the added predictive value of limb praxis measures by comparing 2 nested models—1 including and 1 excluding limb praxis tasks; the results of the ANOVA model comparison are detailed below.

## Results

Our step-wise linear regression model identified 60% of the variance in 9-month BI-ADL scores to be significantly explained by patients’ early subacute performance on both the ADL at baseline and on specific praxis and cognitive measures (F[15 230]=25.48; *P*<0.001; adjusted R2, 0.59). ADL change was best predicted by ADL at early subacute stage (*P*<2×10^−16^; *T*=−16.146; β estimate, −0.626081; SE=0.038776). Interestingly cognitive measures of limb praxis predominantly including gesture production (or pantomime *P*=0.000813; *T*=−3.393; β estimate=−0.055532; SE, 0.016365), gesture recognition (*P*=0.017076; *T*=−2.403; β estimate, −0.034875; SE=0.014516), and meaningless gesture imitation (*P*=0.004673; *T*=2.857; β estimate, 0.033833; SE=0.011844) were important predictors. The slope of association between each of these praxis measures and ADL change scores is displayed in the Figure. Additional cognitive predictors includes orientation in time and space (*P*=0.002796; *T*=−3.022; βestimate, −0.052508; SE=0.017376), measures of language production—including word and nonword reading (*P*=0.009295; *T*=2.623; β estimate, 0.020205; SE, 0.007702), as well as sentence construction (*P*=0.001568; *T*=−3.200; β estimate, −0.040970; SE, 0.012803); and finally tactile extinction to the left during bilateral stimulation (*P*=0.034991; *T*=2.121; β estimate, 0.015937; SE=0.007514).

**Figure.**
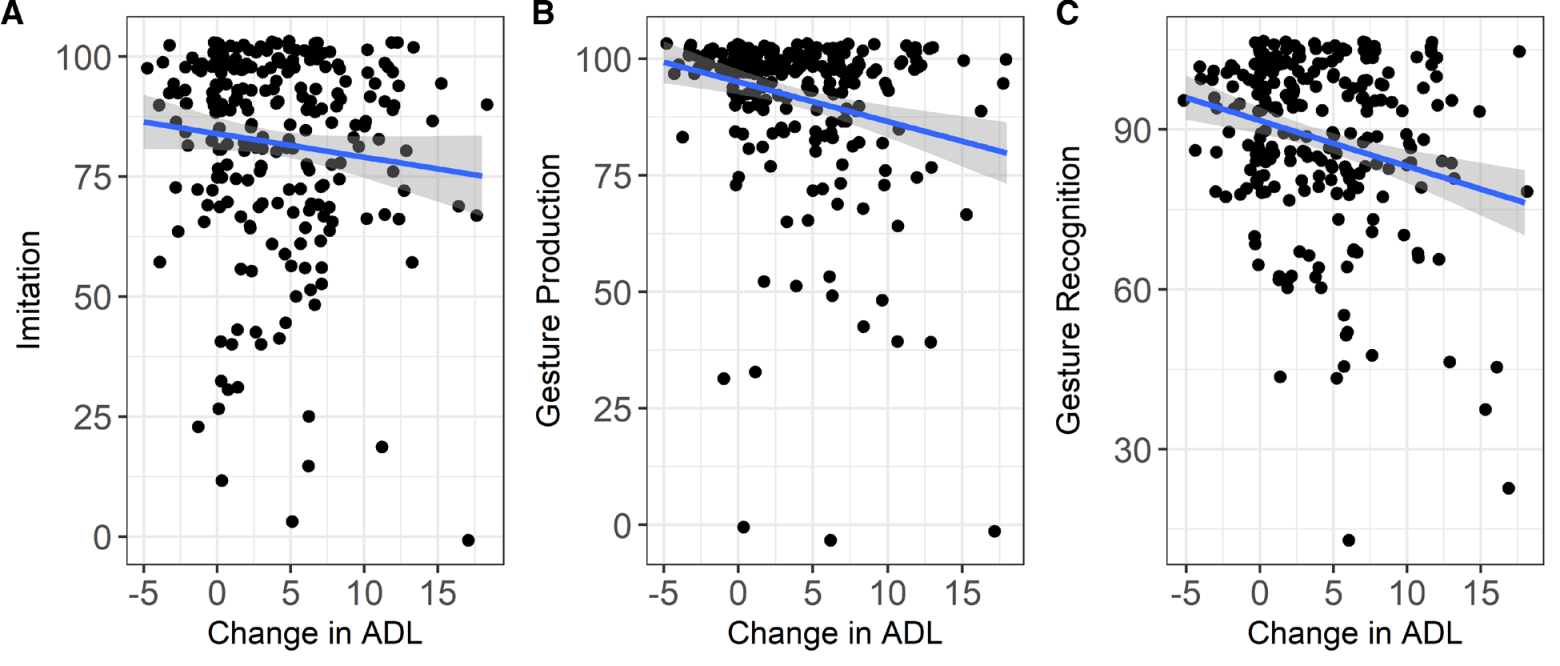
**Associations between early subacute praxis performance and change in activities of daily living (ADL).** Each shows a linear fit (solid line) with 95% CI (shaded) for the relation between Barthel index chronic (ΔBI) ADL–subacute, range (0–20) and (**A**) meaningless gesture imitation (%), (**B**) gesture production (%), and (**C**) gesture recognition (%). Points are jittered for visibility. Praxis scores are scaled 0 to 100.

To assess the significance of limb praxis scores in these predictions, we repeated a stepwise linear regression model excluding the limb praxis scores and an ANOVA comparing the model with and the 1 without the scores (shown in the Supplemental Material; Table S4). The model without apraxia accounted for 56% of the variance (F[11 234]=29.4; *P*<2×10^−^^16^; adjusted R2, 0.56). An ANOVA revealed a significant difference between the 2 models, suggesting that presence of apraxia measures outlined above was significant in predicting BI-ADL change (F=6.8; sum of square, 220.92; *P*<3.544×10^−^^5^ shown in the Supplemental Material; Table S5).

## Discussion

Neurobehavioral deficits after stroke are now recognized as having a profound impact on reintegration into prestroke social and occupational roles, significantly limiting meaningful participation in ADL.^12^ Using data from a unique longitudinal stroke patient data set, namely BCoS, this study provides evidence that detailed cognitive measures, including but not limited to apraxia measures, contribute to predicting these outcomes.

Our results thus extend previous suggestions that limb praxis may be related to patient outcomes (2, 3). We showed that the use of an extended screen involving several limb praxis and other cognitive tasks delivered at subacute stages after stroke could be predictive of patients’ ability to recover on measures of daily living activities at the chronic stage, taking into account their initial scores in daily activities. We identified 8 cognitive measures that were predictive of ADL recovery, reinforcing the need for a comprehensive cognitive screening approach rather than an exclusive focus on limb praxis. This is most important in view of the recognition of a large variability in cognitive screening tools used poststroke in which the most relevant patients’ deficits may not fully be captured. Additionally, the limitation of combining these with outcome measures has only recently been recognized. The BCOS has, therefore, provided us with a unique data set comprising praxis, which is not routinely being collected when patients leave stroke units.

The OCS (Oxford Cognitive Screening) program is the only other study that has provided a comprehensive (yet briefer) cognitive poststroke longitudinal assessment comprising cognitive tasks along with outcome measures. However, this study only comprised 1 praxis task (meaningless gesture imitation) and only tested patients up to 6 months.^13^ It found high early apraxia prevalence (70%), declining by 6 months, with persistent deficits linked to worse ADLs.^13^ However, OCS lacked comprehensive apraxia testing and defined chronic stage at just 6 months. Since apraxia recovery can take 2 to 8 months,^14^ our >9-month follow-up better captures recovery and ADL prediction. Recovery from apraxia has been reported to take between 2 and 8 months poststroke, with some patients recovering faster depending on the praxis tasks involved (5, 14). Hence, a follow-up of over 9 months, as the 1 presented here, would more accurately capture those that have recovered, thus enabling predictions about change in ADL based on praxis and cognitive measures at early subacute stages.

Despite its importance, limb apraxia is frequently undertested in stroke populations. One possible reason for this omission is the historical emphasis on language and motor impairments in early poststroke cognitive testing, with praxis deficits often being overlooked or attributed to general motor dysfunction. Additionally, standardized limb praxis testing is less widely implemented in clinical settings, possibly due to time constraints or a lack of awareness regarding its impact on functional recovery. Ensuring that limb praxis is assessed along with other cognitive functions before discharge could help identify patients at greater risk of persistent ADL difficulties.

This study has limitations. Although we identified praxis as a relevant predictor of ADL outcomes, the sensitivity and specificity of different praxis measures in detecting impairments that strongly correlate with ADL recovery remain to be fully explored. Initial praxis scores were relatively high in our cohort, raising the question of whether certain thresholds of impairment at baseline are particularly indicative of long-term difficulties. We chose regression on ADL change rather than mixed effects models due to the structure of our data (2 assessment time points per participant). We were unable to perform an out-of-sample validation of our results due to a lack of data sets with comprehensive cognitive, specifically detailed praxis, assessments combined with outcome measures collected at early and late stages poststroke. Future research should examine the predictive value of specific cutoff scores in several large cohorts involving longer follow-up periods to guide clinical decision-making. Nevertheless, our findings underscore the critical value of appropriate testing of cognitive and praxis functions such as that provided by BCOS in advancing our understanding of cognitive predictors of poststroke recovery.

## Article Information

### Acknowledgments

The author would like to thank the patient participants taking part in the Birmingham Cognitive study, as well as Ee Lynn Teo for her help with the graphical abstract provided.

### Sources of Funding

Dr Rounis is funded by a United Kingdom Research and Innovation Clinical Academic Partnership program. Dr Demeyere is supported by an Advanced Fellowship from the National Institute for Health and Care Research (NIHR) and project grant from the Stroke Association. Dr Bickerton was an original member of the BCoS study (Birmingham Cognitive Screen), which was funded by the NIHR. Professor Lambon Ralph is supported by a Medical Research Council Intramural Grant.

### Disclosures

None.

### Supplemental Material

Tables S1–S5

Figure S1

STROBE Checklist

## Supplementary Material
