# Supplementary material for: Apraxia as a Predictor of Poststroke Recovery: Insights From the Birmingham Cognitive Screening Program
Source: Stroke. 2025 Oct 7;56(12):3522–6. doi: 10.1161/STROKEAHA.125.051414 (PMC12643566; doi:10.1161/STROKEAHA.125.051414)
Supplement: Supplementary file 1 [file str-56-3522-s001.pdf]

STROBE Statement—checklist of items that should be included in reports of observational studies

	Item No.	Recommendation	Page No.	Relevant text from manuscript
**Title and abstract**	1	(*a*) Indicate the study's design with a commonly used term in the title or the abstract		2 Abstract
		(*b*) Provide in the abstract an informative and balanced summary of what was done and what was found		2 Abstract
**Introduction**				
Background/rationale	2	Explain the scientific background and rationale for the investigation being reported		3 Introduction(1st 3 paragraphs)
Objectives	3	State specific objectives, including any prespecified hypotheses		5 Abstract and Introduction last 3 paragraphs
**Methods**				
Study design	4	Present key elements of study design early in the paper		3-4 **Abstract** Methods**;** Statistical Analyses**.**
Setting	5	Describe the setting, locations, and relevant dates, including periods of recruitment, exposure, follow-up, and data collection		4 **Participants & Measures** (UK, multi-centre; 2010–2015; subacute <1 month & chronic >9 months).
Participants	6	(*a*) *Cohort study*—Give the eligibility criteria, and the sources and methods of selection of participants. Describe methods of follow-up<br>*Case-control study*—Give the eligibility criteria, and the sources and methods of case ascertainment and control selection. Give the rationale for the choice of cases and controls<br>*Cross-sectional study*—Give the eligibility criteria, and the sources and methods of selection of participants		4-5 **Participants & Measures** (first-ever CT-confirmed stroke; consent; exclusions; two time points).
		(*b*) *Cohort study*—For matched studies, give matching criteria and number of exposed and unexposed<br>*Case-control study*—For matched studies, give matching criteria and the number of controls per case		Not applicable
Variables	7	Clearly define all outcomes, exposures, predictors, potential confounders, and effect modifiers. Give diagnostic criteria, if applicable		5 **Neuropsychological**

			**Assessments** (34 BCoS tasks; 4 praxis; constructional praxis); **Outcome** BI-ADL (20-point).
Data sources/ measurement	8*	For each variable of interest, give sources of data and details of methods of assessment (measurement). Describe comparability of assessment methods if there is more than one group	*5* **Neuropsychological Assessments** (published criteria; videotaped; 0–100 standardisation); **Participants & Measures** (trained raters, standardised protocols).
Bias	9	Describe any efforts to address potential sources of bias	3 and 7 **Participants & Measures** (multi-centre recruitment; blinding to outcomes); **Supplement** Tables S1–S3 (comparison of included vs excluded).
Study size	10	Explain how the study size was arrived at	6 **Methods** (sample size/power paragraph using *pwrss*

Continued on next page

Quantitative variables	11	Explain how quantitative variables were handled in the analyses. If applicable, describe which groupings were chosen and why	5 **Neuropsychological Assessments** (0–100 scaling); **Statistical Analyses** (continuous predictors; ADL change).
Statistical methods	12	(*a*) Describe all statistical methods, including those used to control for confounding	5 **Statistical Analyses** (stepwise multiple linear regression predicting chronic BI with baseline BI and cognitive domains; 4-fold CV).
		(*b*) Describe any methods used to examine subgroups and interactions	Not applicable (not performed).
		(*c*) Explain how missing data were addressed	**Supplement** Tables S1–S3 & Figure S1 (flow & missingness per STROBE).
		(*d*) *Cohort study*—If applicable, explain how loss to follow-up was addressed<br>*Case-control study*—If applicable, explain how matching of cases and controls was addressed<br>*Cross-sectional study*—If applicable, describe analytical methods taking account of sampling strategy	**Supplemental Material** Figure S1 (424 recruited → 256 analysed; reasons/attrition)**.**
		(*e*) Describe any sensitivity analyses	**Statistical Analyses** (nested models with/without praxis; ANOVA) and **Results**; **Supplement** Tables S4–S5.
**Results**			
Participants	13*	(a) Report numbers of individuals at each stage of study—eg numbers potentially eligible, examined for eligibility, confirmed eligible, included in the study, completing follow-up, and analysed	**Supplement** Figure S1 (flowchart).
		(b) Give reasons for non-participation at each stage	**Supplement** Figure S1 / tables
		(c) Consider use of a flow diagram	**Supplement** Figure S1 (excluded box to the right).
Descriptive data	14*	(a) Give characteristics of study participants (eg demographic, clinical, social) and information on exposures and potential confounders	7 **Table 1** (main text).
		(b) Indicate number of participants with missing data for each variable of interest	"Complete case" in main text; details in **Supplement** Table S3 (by

			task/category).
		(c) *Cohort study*—Summarise follow-up time (eg, average and total amount)	7 **Follow-up time: Table 1** (avg days since stroke at both time points).
Outcome data	15*	*Cohort study*—Report numbers of outcome events or summary measures over time	9 **Results** (BI baseline 13.3±5.5 → 17.3±3.9 at >9 months).
		Case-control study—Report numbers in each exposure category, or summary measures of exposure	
		Cross-sectional study—Report numbers of outcome events or summary measures	
Main results	16	(*a*) Give unadjusted estimates and, if applicable, confounder-adjusted estimates and their precision (eg, 95% confidence interval). Make clear which confounders were adjusted for and why they were included	10 **adjustments: Table 2** and **Results** (betas, SEs, p-values; model adjustment incl. baseline BI).
		(*b*) Report category boundaries when continuous variables were categorized	Not applicable (continuous predictors retained).
		(*c*) If relevant, consider translating estimates of relative risk into absolute risk for a meaningful time period	Not applicable.

Continued on next page

Other analyses	17	Report other analyses done—eg analyses of subgroups and interactions, and sensitivity analyses	8-9 **Results**; **Supplement** Tables S4–S5 (model without praxis; ANOVA).
**Discussion**			
Key results	18	Summarise key results with reference to study objectives	9 **Discussion** (opening paragraph).
Limitations	19	Discuss limitations of the study, taking into account sources of potential bias or imprecision. Discuss both direction and magnitude of any potential bias	10 **Discussion** (limitations; two-time-point design; lack of external dataset).
Interpretation	20	Give a cautious overall interpretation of results considering objectives, limitations, multiplicity of analyses, results from similar studies, and other relevant evidence	10 **Discussion** (context vs OCS, prior evidence).
Generalisability	21	Discuss the generalisability (external validity) of the study results	10 **Discussion** (multi-centre UK; comparison of included vs excluded; STROBE flow).
**Other information**			
Funding	22	Give the source of funding and the role of the funders for the present study and, if applicable, for the original study on which the present article is based	12 **Sources of Funding** (spelled-out agencies per journal request). No funder role asserted.

*Give information separately for cases and controls in case-control studies and, if applicable, for exposed and unexposed groups in cohort and cross-sectional studies.

Note: An Explanation and Elaboration article discusses each checklist item and gives methodological background and published examples of transparent reporting. The STROBE checklist is best used in conjunction with this article (freely available on the Web sites of PLoS Medicine at http://www.plosmedicine.org/, Annals of Internal Medicine at http://www.annals.org/, and Epidemiology at http://www.epidem.com/). Information on the STROBE Initiative is available at www.strobe-statement.org.
